# Bioenergetic functions in subpopulations of heart mitochondria are preserved in a non-obese type 2 diabetes rat model (Goto-Kakizaki)

**DOI:** 10.1038/s41598-020-62370-8

**Published:** 2020-03-25

**Authors:** N. Lai, C. M. Kummitha, F. Loy, R. Isola, C. L. Hoppel

**Affiliations:** 1 Department of Electrical and Computer Engineering, Old Dominion University, Norfolk, Virginia USA; 2 Department of Biomedical Engineering Institute, Old Dominion University, Norfolk, Virginia USA; 3 Department of Biomedical Engineering, School of Medicine, Case Western Reserve University, Cleveland, USA; 4 Department of Pharmacology, School of Medicine, Case Western Reserve University, Cleveland, USA; 5 Center for Mitochondrial Disease, School of Medicine, Case Western Reserve University, Cleveland, USA; 6 Department of Medicine, School of Medicine, Case Western Reserve University, Cleveland, USA; 7 Department of Mechanical, Chemical, and Materials Engineering, University of Cagliari, Cagliari, USA; 8 Department of Biomedical Sciences, University of Cagliari, Cagliari, USA

**Keywords:** Biochemistry, Cardiovascular diseases

## Abstract

A distinct bioenergetic impairment of heart mitochondrial subpopulations in diabetic cardiomyopathy is associated with obesity; however, many type 2 diabetic (T2DM) patients with high-risk for cardiovascular disease are not obese. In the absence of obesity, it is unclear whether bioenergetic function in the subpopulations of mitochondria is affected in heart with T2DM. To address this issue, a rat model of non-obese T2DM was used to study heart mitochondrial energy metabolism, measuring bioenergetics and enzyme activities of the electron transport chain (ETC). Oxidative phosphorylation in the presence of substrates for ETC and ETC activities in both populations of heart mitochondria in T2DM rats were unchanged. Despite the preservation of mitochondrial function, aconitase activity in T2DM heart was reduced, suggesting oxidative stress in mitochondria. Our study indicate that metabolic function of heart mitochondria is unchanged in the face of oxidative stress and point to a critical role of obesity in T2DM cardiomyopathy.

## Introduction

Patients with type 2 diabetes mellitus (T2DM) are vulnerable to heart disease and have a two-fold risk for several vascular diseases^1^. Heart failure is the main cause of death in 65% of the diabetic population, highlighting the need to understand the causes of diabetic cardiomyopathy^2^. Metabolic abnormalities in diabetic hearts contribute to the development of impaired contractility observed in diabetic-related cardiomyopathies^3,4^. In particular, dysfunction of mitochondrial bioenergetics has been related to the pathogenesis of diabetic cardiomyopathy^5^, as heart contraction depends mostly on ATP produced by the mitochondrial oxidative phosphorylation system^4^.

Reduced cardiac efficiency and mitochondrial energetics, increased fatty acid oxidation, and increased lipid content occur in both obese and type 2 diabetic patients^6^. These cardiac abnormalities have been associated with obesity and T2DM, but it is not clear whether mitochondrial alterations were strictly related to obesity or insulin resistance. A relationship between obesity and mitochondrial dysfunction has been established^7^ in human heart. In that study, enzymatic measurements in right atrial tissue showed a reduced complex I activity of the electron transport chain in young and old obese patients in comparison to young and old healthy control groups^7^. In contrast, impairment of myocardial contractility function has been associated with mitochondrial dysfunction in T2DM rather than in obese patients^5^. Positron emission tomography studies^8,9^ on human heart are consistently indicating that both obesity and insulin resistance are contributing to an alteration of heart substrate utilization. In addition to the PET studies, magnetic resonance spectroscopy^10^ studies provide evidence for a correlation between diastolic dysfunction and cardiac triglyceride levels which are higher in healthy obese subjects and lean and obese diabetic patients than lean healthy subjects^11,12^.

Cardiac abnormalities in obese and type 2 diabetic patients have been investigated with animal models of obesity and type 1 diabetes (T1DM) and T2DM^6^ in which cardiac contractile efficiency and mitochondrial metabolism showed progressive declines^13,14^ with an increased reactive oxygen species (ROS) production and lipid peroxidation. Although both model of type 1 and 2 are used to study diabetic cardiomyopathy, differences in bioenergetic function exist between them. In T1DM mice fed a regular chow diet, cardiac dysfunction was reported without any mitochondrial respiration defects, but in T2DM mice fed with high-fat diet, insulin resistance was accompanied by impairment of oxidative phosphorylation^5^. Nevertheless, these studies did not investigate the subpopulations of heart mitochondria (subsarcolemmal and interfibrillar), which have been reported to be differently affected by cardiomyopathy in hamster^15^ and mice with T1DM^16^ and T2DM^17^.

Among the animal models^18^ of diabetic cardiomyopathy, Goto-Kakizaki (GK) rats^6,19^ have the unique feature of being insulin-resistant without obesity^20,21^. The GK model was reported to have a mild cardiomyopathy characterized by diastolic dysfunction^20^. Increased susceptibility to oxidative stress was observed in GK heart mitochondria^22^, but bioenergetic functions were not reported.

A previous study in skeletal muscle of GK rats showed preserved bioenergetic function in both mitochondrial subpopulations^23^. In the current study, we evaluated bioenergetic function in heart mitochondrial subpopulations of the same non-obese diabetic GK rats at 18 and 28 weeks and found that metabolic function is preserved in both subpopulations of mitochondria despite induced mitochondrial stress.

## Results

### Animal model

The animal model characteristics are reported in Table 1. The body and heart weight of diabetic (GK) rats is significantly reduced in comparison to the control (W) rats at both 18 and 28 weeks. The body and heart weight of GK rats does not change from 18 to 28 weeks, while those of W rats significantly increases by 24%^23^ and 28%, respectively. The GK rats are hyper-insulinemic and hyperglycemic at 18 and 28 weeks.

**Table 1:** Animal characteristics: body^23^ and heart weight, insulin and glucose concentrations^23^ in blood.

	Unit	Wistar	GK	Wistar	GK
		18 wk		28 wk	
Body weight	[g]	474 ± 47	350 ± 23*	590 ± 58^#^	389 ± 21*
Heart weight	[g]	0.97 ± 0.1	0.8 ± 0.09*	1.24 ± 0.13^#^	0.81 ± 0.06*
Insulin	[ng mL^−1^]	2.8 ± 1.4	5.9 ± 1.6*	2.4 ± 2.1	5.5 ± 1.1*
Glucose	[mM]	6 ± 1	16.5 ± 2.3*	6.5 ± 1.7	17.4 ± 3*

Data are mean ± SD (n = 6).

Influence of insulin resistance within same age (p < 10^−3^): (*) Statistically different from control.

Influence of age within group (p < 10^−5^): (^#^) Statistically different from 18 weeks.

**Figure 1:**
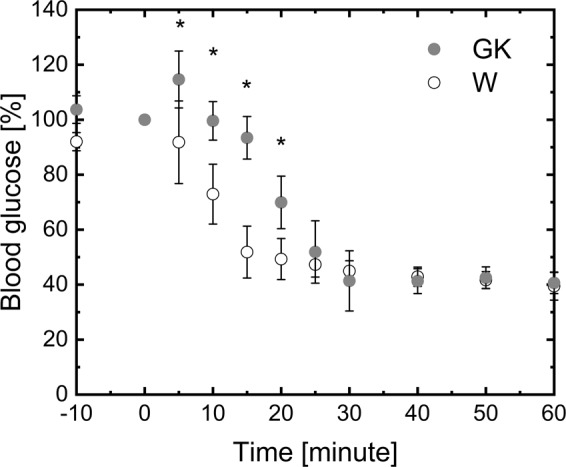
Time profile of blood glucose content after bolus injection of insulin. Insulin tolerance tests is performed on random-fed control (Wistar) and diabetic (GK) rats at 12 weeks. Animals are injected subcutaneously with human regular insulin (1.85 U/kg body weight). Blood glucose is measured before and after injection (n = 4). The basal glucose concentration of Wistar and GK rats is 7.5 ± 0.6 and 12.6 ± 0.4 mM, respectively (n = 4). Mean ± SD. (^*^) Statistically different from Wistar.

For both group of rats, the insulin tolerance test with the time profile of blood glucose concentrations is reported in Fig. 1. Blood glucose concentration is normalized to the basal blood glucose concentration measured before insulin injection. In the first 20 minutes of the test, the normalized blood glucose content in GK was significantly higher than that observed for W. The basal glucose concentration of W and GK was 7.5 ± 0.6 and 12.6 ± 0.4 mM.

### Mitochondrial yield and enzymes

The yield of SSM and IFM did not differ in GK and W rats or with age (Table 2). CS and SDH activities were similar in GK and W rats at 18 and 28 wk. At 18 wk, the activity of aconitase was significantly reduced in both subpopulations of mitochondria of GK rats in comparison to the control group (Table 2), whereas at 28 wk in IFM, it was significantly lower in GK than W; there is a trend toward significance between SSM GK and W rats.

**Table 2:** Yields and enzyme activities of subsarcolemmal (SSM) and interfibrillar (IFM) mitochondria.

		W	GK	W	GK
		18 wk		28 wk	
	**Mitochondrial Yield [mg gww**^**−1**^**]**				
	**SSM**	16.7 ± 2.1	17.8 ± 5.3	14.4 ± 2.4	15.2 ± 1.5
	**IFM**	23.1 ± 2.2	23.0 ± 6.1	20.7 ± 1.2	18.8 ± 2.3
	**Isolated mitochondria [mU mg**^**−1**^**]**^**a**^				
Citrate Synthase	**SSM**	1912 ± 198	2123 ± 263	2151 ± 353	2310 ± 305
	**IFM**	2365 ± 195	2284 ± 406	2742 ± 213	2745 ± 364
Succinate Dehydrogenase	**SSM**	297 ± 38	304 ± 33	328 ± 36	349 ± 30
	**IFM**	361 ± 28	313 ± 57	393 ± 20	384 ± 19^#^
Aconitase	**SSM**	761 ± 132	542 ± 107*	845 ± 51	700 ± 68
	**IFM**	803 ± 174	489 ± 75*	1056 ± 112^#^	797 ± 80*^,#^

^a^The mitochondrial yields and enzymatic activity of the subpopulations of heart mitochondria is normalized to gram of wet weight of heart muscle (gww) and mitochondrial protein (mg), respectively. Data are mean ± SD (n = 6).

Influence of insulin resistance within same age (p < 5 10^−3^): (^*^) Statistically different from Wistar.

Influence of age within group (p < 5 10^−3^): (^#^) Statistically different from 18 weeks.

### Immunoblotting

**Figure 2:**
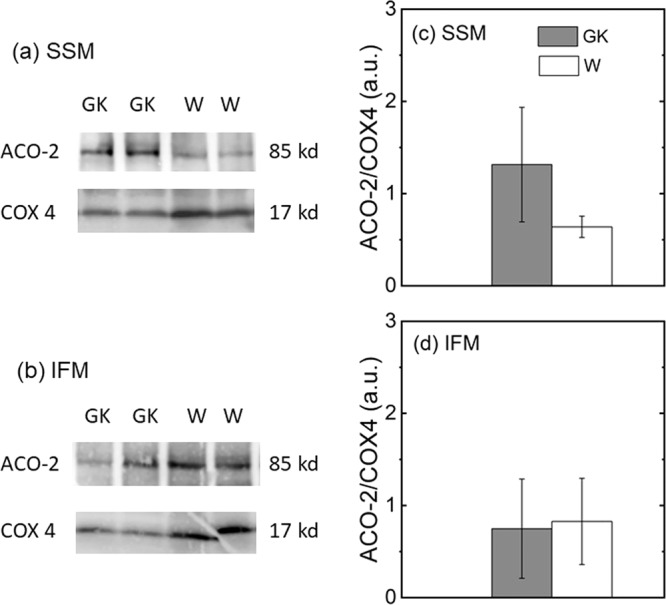
Immunoblotting of aconitase (ACO-2) protein in heart muscle SSM and IFM at 28 weeks. Control (W) and diabetic (GK) groups are represented with open and grey bars, respectively. Representative immunoblotting for SSM (**a**) and IFM (**b**) and densiometric analysis of SSM (**c**) and IFM (**d**) of ACO-2 protein. Data are normalized to the intensity obtained with the housekeeping gene COX4. (n = 4–5). Full-length western blots are presented in Supplementary Fig. S2. Data are mean ± SD.

To confirm that the expression of mitochondrial aconitase was unaltered in our T2DM lean diabetic model, we determined ACO-2 (aconitase) content by immunoblotting in subpopulation of mitochondria isolated from rat hearts at 28w (Fig. 2). In both SSM and IFM the ACO-2 expression was not different in GK rats as compared to W rats, despite a tendency to be higher in GK SSM, albeit not statistically significant.

### Oxidative phosphorylation

**Figure 3:**
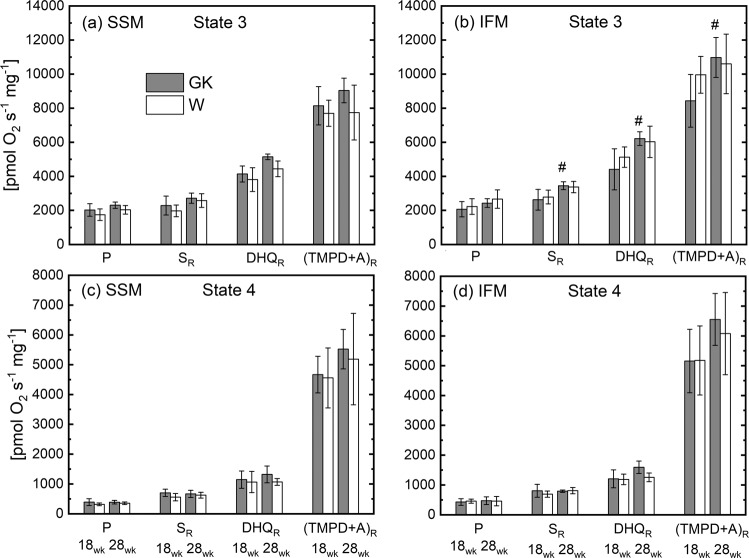
State 3 (**a**,**b**) and State 4 (**c**,**d**) respiration rates of heart muscle SSM and IFM at 18 and 28 weeks. Notation as in Fig. 2. Complex I substrate (malate and pyruvate, P); Complex II (succinate and rotenone, S_R_); Complex III (duroquinol and rotenone, DHQ_R_); Complex IV (TMPD, ascorbate and rotenone, (TMPD + A)_R_. (n = 6), Mean ± SD. (^#^) Within the group statistically different from 18 weeks.

**Figure 4:**
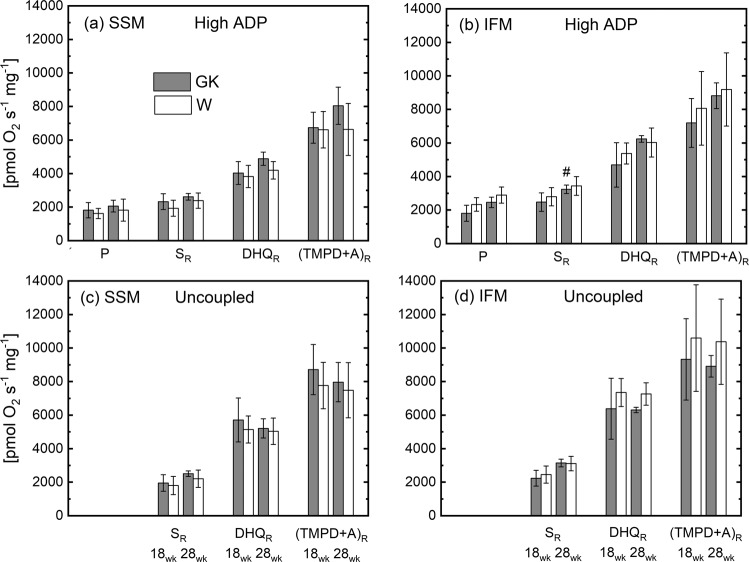
High ADP concentration (**a**,**b**) and uncoupled (**c**,**d**) respiration rates of heart muscle SSM and IFM at 18 and 28 weeks. Notation as in Fig. 2. Complex I substrate (malate and pyruvate, P); Complex II (succinate and rotenone, S_R_); Complex III (duroquinol and rotenone, DHQ_R_); Complex IV (TMPD, ascorbate and rotenone, (TMPD + A)_R_. (n = 6), Mean ± SD. (^#^) Statistically different from 18 weeks.

Oxidative phosphorylation for heart muscle SSM and IFM was measured using substrates for complex I (glutamate Table 3), I, II, III, and IV in both GK and W rats (Figs. 3 and 4) at 18 and 28 weeks. The state 3 and 4 respiratory rates in SSM were similar in the GK rats compared to those in the W (Table 3). Also, in GK and control rats at both ages, SSM respiration rates obtained with a saturated concentration of ADP increased ~20% and were comparable (Fig. 4), indicating that ADP under state 3 conditions is not saturating. With the addition of an uncoupler, the respiratory rates did not increase, indicating that oxidative phosphorylation is limited by oxidation in both groups of rats. In the IFM of both groups of rats, the results were similar to those obtained for SSM. Thus, in this model of T2DM oxidative phosphorylation rates were unaffected by insulin resistance (Figs. 3 and 4).

**Table 3:** Oxidative phosphorylation using glutamate in heart muscle subsarcolemmal mitochondria (SSM) and interfibrillar mitochondria (IFM) in control (W) and diabetic (GK) rats.

	Unit	W	GK	W	GK
		18 wk		28 wk	
**SSM**					
**State 3**	[pmolO_2_ s^−1^ mg^−1^]	1723 ± 301	2041 ± 218	2035 ± 197	2244 ± 143
**State 4**	[pmolO_2_ s^−1^ mg^−1^]	160 ± 18	200 ± 48	150 ± 31	157 ± 35
**RCR**	[-]	11 ± 3	11 ± 2	14 ± 3	15 ± 5
**ADP/O**	[-]	3.07 ± 0.19	2.86 ± 0.34	2.82 ± 0.2	3.03 ± 0.27
**High ADP**	[pmolO_2_ s^−1^ mg^−1^]	2177 ± 255	2554 ± 341	2257 ± 229	2647 ± 222
**DNP**	[pmolO_2_ s^−1^ mg^−1^]	1942 ± 268	2513 ± 320	2273 ± 329	2607 ± 357
**IFM**					
**State 3**	[pmolO_2_ s^−1^ mg^−1^]	2259 ± 231	2026 ± 430	2490 ± 189	2551 ± 200^#^
**State 4**	[pmolO_2_ s^−1^ mg^−1^]	219 ± 67	198 ± 47	185 ± 33	156 ± 41
**RCR**	[-]	11 ± 5	10 ± 1	14 ± 3	18 ± 6
**ADP/O**	[-]	2.99 ± 0.16	2.99 ± 0.24	3.11 ± 0.5	3.05 ± 0.3
**High ADP**	[pmolO_2_ s^−1^ mg^−1^]	2821 ± 345	2647 ± 609	2890 ± 305	3042 ± 211
**DNP**	[pmolO_2_ s^−1^ mg^−1^]	2767 ± 379	2512 ± 580	2922 ± 493	3191 ± 343

The respiratory rate is normalized to the content (i.e. mg) of heart mitochondrial protein. (^#^) Statistically different from 18 weeks.

The state 3 respiration rate obtained with saturated concentration of ADP (i.e. High ADP, Table 3), was significantly correlated to the activity of CS (r^2^ = 0.63, p < 10^−5^) or SDH (r^2^ = 0.65, p < 10^−5^) with a slope of the linear relationship significantly different from zero in both cases. The RCR of both groups was higher than 10, indicating that both populations of mitochondria are highly coupled. The ADP/O ratio is the same for both populations of mitochondria (Table 3). The ADP/O ratios determined for complexes I, II, and III substrates were similar in GK and W rats for both subpopulations of mitochondria (Fig. S1).

### Fatty acid oxidation

**Figure 5:**
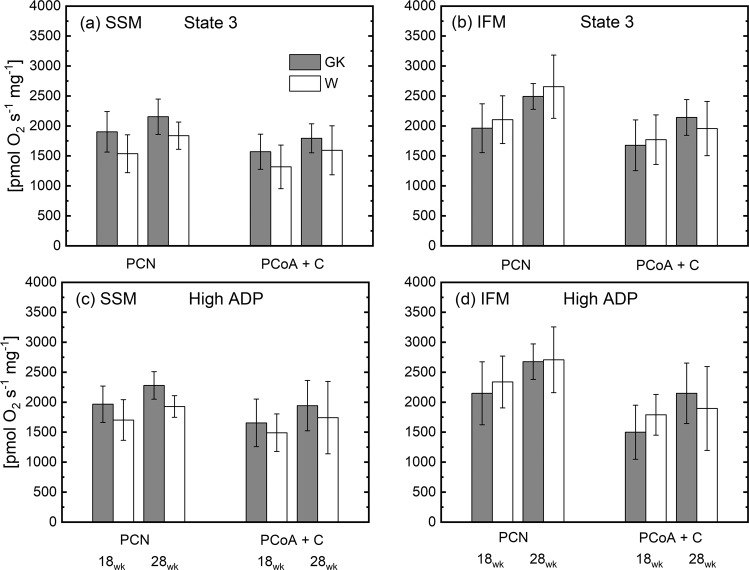
ADP unsaturated (**a**,**b**) and saturated concentration (**c**,**d**) respiratory rates of lipid substrates in heart muscle SSM and IFM at 18 and 28 weeks. Notation as in Fig. 2. Malate and palmitoylcarnitine, PCN; malate, palmitoyl-CoA and carnitine (P-CoA + C).

The respiration rate of SSM and IFM are measured in the presence of a long-chain fatty acid (FA) substrates: palmitoylcarnitine (PCN) or palmitoyl-CoA (PCoA) to study mitochondrial fatty acid oxidation. In both subpopulations of mitochondria, fatty acid oxidation in the presence of malate for both PCN or PCoA was similar in GK and W rats (Fig. 5) at 18 and 28 wk.

### Electron transport chain

**Figure 6:**
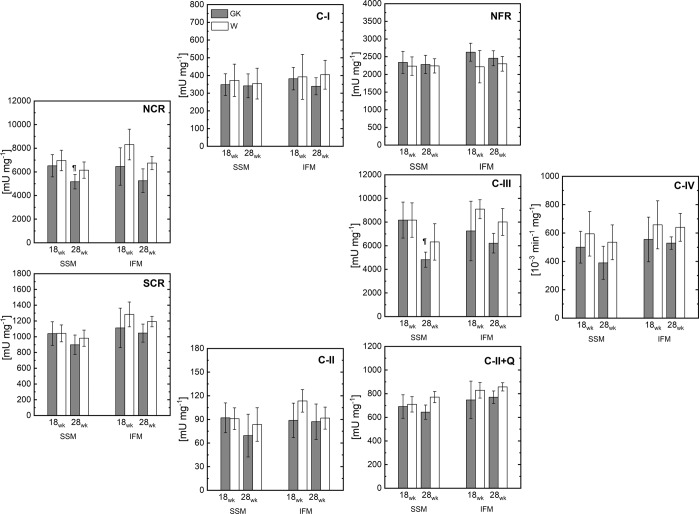
The enzymes activity of ETC of isolated heart SSM and IFM at 18 and 28 weeks. Rotenone-sensitive NADH-cytochrome c reductase (NCR); NADH ferricyanide reductase (NFR); Antimycin A-sensitive succinate-cytochrome c reductase (SCR); Complex II activity (CII); total complex II with exogenous coenzyme Q (CII + Q); Complex III (CIII); Complex IV (CIV). ^¶^(P < 0.05) GK-18wk vs. GK-28wk; (n = 6) Mean ± SD. (^#^) Within the group statistically different from 18 weeks.

The activity of the ETC complexes was evaluated with specific spectrophotometric assays. In SSM and IFM of GK rats, the activity of the ETC components of a) complex I, II, III, and IV; b) linked complex I and III (NCR), c) flavin protein domain of complex I (NFR); d) linked complex II and III (SCR); e) complex II (SDH) were similar to those of the W (Fig. 6) at 18 and 28 wk.

## Discussion

This study focused on subpopulations of heart mitochondria of GK rats to compare their bioenergetics with that of control rats at 18 and 28 weeks. Heart mitochondria were obtained from the same rats investigated in a previous study on skeletal muscle mitochondria^23^.

In SSM and IFM heart mitochondria of non-obese diabetic rats (GK) bioenergetic function was similar to the control group both at the age of 18 and 28 weeks. This was true with substrates for complex I, II, III, and IV, as well as of fatty acid oxidation. Electron transport chain activities were unchanged in GK confirming that mitochondrial bioenergetic function was preserved. The reduced aconitase activity in GK heart mitochondrial subpopulations indicates that despite the presence of oxidative stress, the bioenergetic function was preserved.

### Animal model

In our work, GK rats were resistant to a decrease in blood glucose level after a bolus injection of insulin (Fig. 1). The delayed effects of the administered insulin on glucose levels in blood suggested the presence of insulin resistance. Also, the hyperglycemia and hyperinsulinemia observed in the GK rats at both age groups confirmed the metabolic characteristics of this T2DM model. In previous GK rat studies, skeletal^24,25^ and heart^26^ muscle as well as liver^27^ and adipose tissue^28^ were insulin-resistant.

The GK rat exhibits spontaneous moderate hyperglycemia hyperinsulinemia and high plasma triglyceride levels^21,29^ without abnormal elevated content of non-esterified fatty acids (NEFA). GK hearts have been consistently reported to be hypertrophied^26,29^, elevated cardiac NEFA and triglycerides with alteration of cardiac structure and function^30^. Thus, there are several evidence supporting that the GK rats represent a reliable model of diabetic cardiomyopathy^29^ in absence of obesity.

The reduced heart weight in diabetic rats was also found in another study on GK hearts^20^ with a similar age to our group. These findings are consistent with another rat model of cardiomyopathy in which the reduced mass was attributed to the lack of insulin on heart myocyte growth and protein synthesis^31^. In contrast, GK male rats at 47 weeks were reported to have similar heart weight to the control group^26^. The difference between our group and that of the previous study appears to be age related because the rats of our study were several months younger (28 weeks). Indeed, GK heart weight were reported to be increased, decreased or unaltered for different age groups^29,32^.

### Mitochondria function

Cardiac mitochondrial dysfunction has been reported in obese subjects with^33^ and without insulin resistance^7^, but the contribution of hyperglycemia and obesity to this dysfunction remains to be determined^34^. Also, these reports are relevant for T2DM patients who are not obese and who are regarded to be at high risk for cardiovascular disease^35,36^. An age-dependent relationship between cardiomyopathy and heart mitochondrial dysfunction was reported in cardiomyopathic hamsters at 17 and 30 weeks, but not as early as 4 weeks^15^. Thus, we investigated diabetic heart subpopulations of mitochondria in GK rats, a non-obese model of T2DM, at 18 and 28 weeks. The unchanged mitochondrial function and enzymatic activity of the electron transport chain complexes (Fig. 6) are consistent with data of a study on aorta mitochondria of GK rats showing a respiration rate with complex I and β oxidation substrates similar in GK and control rats^37^.

In both group of rats, SSM and IFM respiration rate were similar with a trend to be higher in IFM. This was mainly related to the CS and SDH activities which were similar in both SSM and IFM (Table 2). The effect of the specific activity of mitochondrial marker enzymes (i.e. CS and SDH) on mitochondrial respiratory rate was confirmed by the significant correlation between mitochondrial enzyme marker and respiration rate. In a previous study^38^ on dog heart mitochondria similar respiratory rates in SSM and IFM were accompanied by similar CS activity of SSM and IFM.

Susceptibility of heart SSM and IFM to cardiomyopathy has been shown to be different in obese and insulin resistant animal models. Cardiomyopathy studies with animals without insulin resistance reported dominant dysfunction of IFM in comparison to SSM in heart of hamster^15^ and mouse with T1DM^16,39^. In T1DM mice, mitochondrial respiration with complex I and complex III substrates were reduced only in IFM^16^. In contrast, obese T2DM mice had reduced state 3 respiration rate and electron transport chain activities in SSM with no change in IFM^17^. Also, in heart mitochondria^40^ of T2DM patients, only SSM respiration rate in presence of complex I or FA substrates was compromised, whereas IFM was preserved in obese and insulin resistant patients. Overall, these data suggest that mitochondrial function is spatially sequestered in T2DM heart. Differences associated with animal models and factors such as obesity are determinant of the mitochondrial dysfunction.

### Oxidative stress

Diabetic cardiomyopathy is characterized by alteration of substrate utilization and mitochondrial function accompanied by oxidative stress^41^. A previous GK study reported a high susceptibility of heart mitochondria to lipid peroxidation in the presence of induced oxidative damage^22^, whereas lipid peroxidation products are used as biomarkers of oxidative stress. It has been suggested that the greater susceptibility of diabetic heart mitochondria to oxidative damage was caused by a limited antioxidant potential related to a lower co-enzyme Q and glutathione contents^22,42^, but mitochondria function was not examined. In our study, a reduced mitochondrial aconitase activity in GK rats (Table 2) confirmed the presence of heart mitochondrial oxidative stress in this animal model of T2DM. The iron sulfur cluster of aconitase is sensitive to oxidation by superoxide^43^ which mediates the inactivation of aconitase. Inactivated aconitase is then degraded by Lon protein in the mitochondrial matrix^44^. Thus, the lower aconitase activity is compatible with a higher inactivation rate induced by oxidative stress. Furthermore, a study in 14 week-old GK rats^45^ reported reduced aconitase activity in the aorta and kidney whereas it was unchanged in the heart. In our study, reduced aconitase activity was observed at 18 weeks suggesting a time-dependent process leading to oxidative stress of heart mitochondria that is not evident at 14 weeks. As with our observations at 18 weeks, aconitase decreased activity is present in 28 weeks-old rats, too, confirming that in TDM2 non-obese rats ongoing oxidative stress impairs the function of this important enzyme.

The immunoblotting assay on mitochondrial ACO-2 confirmed a similar aconitase protein expression in mitochondria of both diabetic and control rats (Fig. 2). Thus, it is reasonable to exclude the possibility that the reduced aconitase activity in GK was related to low aconitase expression caused by other factors as, for instance, hypoxia inducible factor which might regulate the aconitase expression^46,47^.

Animal models of obesity and T2DM showed an increase of oxidative stress in the presence of cardiac lipid accumulation with increased fatty acid oxidation^6,33,48^. In our study, myocardial mitochondria enzymatic activity of GK rats suggested the presence of oxidative stress even when the animals were consuming normal diet and not obese. An increase of ROS production could be related to insulin resistance rather than to a myocardial overload^41^; GK rats have been described as myocardial insulin-resistant^19^. In support of this view, in the absence of insulin resistance, a mouse model of T1DM^49^ and patients with type 1 diabetes^50^ did not exhibit oxidative stress. The deleterious interaction of ROS and endoplasmic reticulum stress are suggested to contribute to diastolic dysfunction in diabetic cardiomyopathy^51^, as has been reported in GK rats^20^. Cardiomyopathy observed in GK appears mild but still significantly affects contractility. In the presence of an induced infarction, contractility dysfunction was greater in GK than that in control rats, suggesting that heart failure progression is accelerated in this animal model of T2DM^52^.

An increase of ROS production has been reported to be related to mitochondrial dysfunction due to uncoupling^53^ because superoxide can also be produced as a byproduct of oxidative phosphorylation. In our study, oxidative stress is present in the absence of mitochondrial uncoupling (Table 2). Our data support the view that oxidative stress was not generated by dysfunction of both subpopulations of mitochondria. Previous studies reported that diabetes selectively causes oxidative stress in IFM mitochondria, accompanied by a reduction of respiration, transmembrane potential, and an increase of mitochondrial transition pore opening^16,39^. It is noteworthy that mitochondrial transition pore opening has been suggested to be a consequence of oxidative damage rather than the cause^54^.

Therefore, T2DM did not induce an overt mitochondrial impairment in GK rats, suggesting that in lean insulin-resistant individuals mitochondria retain healthy features. Indeed, it has been shown that heart mitochondria of GK rats are even more resistant to calcium overload than are controls^55^, thus indicating that these mitochondria can better counteract the deleterious effects of diabetes.

In presence of obesity and T2DM conditions mitochondrial dysfunction occurs whereas in the absence of obesity, heart mitochondrial function is unchanged and highlights the critical role for obesity in T2DM. Therefore, our study suggests that insulin resistance does not lead to mitochondrial dysfunction in absence of obesity.

## Materials and Methods

### Animals

A non-obese model of type 2 diabetes mellitus (T2DM), Goto-Kakizaki (GK) rats, and Wistar (W) colony rats (Charles River) as control group were used in this study. GK rats’ manifest spontaneous skeletal muscle and hepatic insulin resistance, mild hyperglycemia, and normal lipidemia. The genetic background of the GK rats is that of Wistar. The GK rats were obtained by selective breeding of Wistar rats with the highest blood glucose levels during an oral glucose tolerance test over many generations^56^.

The insulin tolerance test was performed on regularly fed rats which were not starved overnight prior to the experiment. Experiment was initiated around 9:00AM, rats were not allowed to eat during the course of the experiment. Rats were challenged with a subcutaneous injection of insulin (1.85 U insulin/Kg of body weight). Blood samples from tail vein puncture were obtained at different time points after insulin injection to measure glucose concentration^57,58^.

Twelve male GK and W rats were housed in the Animal Resource Center facilities of Case Western Reserve University with 12:12-h light-dark cycle and were fed a standard diet chow (Prolab Isopro RMH 3000, LabDiet, St. Louis, MO) ad libitum. GK and W rats were euthanized by decapitation at 18 wk (n = 6) and 28 wk (n = 6) of age. All procedures were approved by Case Western Reserve University Institutional Animal Care and Use Committee and performed in accordance with the National Research Council guidelines for care and use of laboratory animals in research. It should be noted that plasma insulin content and bioenergetics of both populations of skeletal muscle mitochondria have been published by our group^23^.

### Buffers

Buffers: the relaxing buffer Chappell-Perry (CP1) (100 mM KCl, 50 mM MOPS, 5 mM MgSO_4_, 1 mM ATP, and 1 mM EGTA^59^), CP2 (Buffer CP1 plus 0.2% defatted BSA) and KME (100 mM KCl, 50 mM MOPS and 0.5 mM EGTA, pH 7.4^60^) were used for isolation and storage of mitochondria. The respiration buffer (80 mM KCl, 50 mM MOPS, 1 mM EGTA, 5 mM KH_2_PO_4_, and 1 mg/mL defatted BSA, pH 7.4^61^) was used for mitochondrial oxygen uptake measurements.

### Mitochondrial isolation

The subsarcolemmal (SSM) and interfibrillar mitochondria (IFM) were isolated from heart muscle as described previously^62^ with modifications including the use of a modified Chappell-Perry buffer^63^ and trypsin to treat myofibrillar pellets^38^. Mitochondrial protein concentration was determined using the Lowry method with bovine serum albumin as standard^64^.

### Oxidative phosphorylation assays

Mitochondrial oxygen consumption was measured using a Clark-type oxygen electrode (YSI model 53) embedded in a glass metabolic chamber containing 0.1–0.25 mg mitochondrial protein in a final volume of 0.5 mL of respiration buffer. The chamber temperature was maintained at 30 °C with a circulating water bath^61^.

The respiration rate of SSM and IFM was measured in the presence of substrates and inhibitors^38^. The assays with complex II, III, and IV substrates were performed with rotenone to inhibit complex I.

Glutamate was used because among the complex I substrates, it is the only substrate that does not require any further additions for the analysis of oxidative substrate. It has its own transporter through the inner membrane and in the matrix it is dehydrogenated by glutamate dehydrogenase yielding NADH, which is then oxidized by complex I of the ETC^65^.

Both low and high ADP concentration were used to stimulate mitochondrial respiration. Because a high ADP concentration does not allow to observe the transition from state 3 to 4 in polarographic systems with O_2_ concentrations near air-saturation, a low ADP concentration was selected to reach state 3 in coupled mitochondria avoiding O_2_ depletion within the chamber during State 4. Also, with saturated ADP concentration (i.e. High ADP), the state 3 respiratory rate is expected to be higher than that obtained with unsaturated concentration because of the specific ADP affinity to adenine nucleotide translocator and mitochondrial enzymes. Thus, in our study, State 4 is equivalent to a leak state (L_T_) with ATP hydrolysis and state 3 indicates the condition under which coupled mitochondria are stimulated with ADP (P)^66^.

Respiratory control ratio (RCR, State 3 divided by State 4) was used to determine the coupling of mitochondrial oxidation and phosphorylation. An enzymatic method^67^ was used to determine the concentration of ADP and AMP for the calculation of the ADP/O ratio (number of ADP moles added for the number of moles of oxygen atom consumed), which is an index of the efficiency of oxidative phosphorylation^68^.

### Preparation of samples and enzymatic assays

Mitochondrial enzyme activities were measured as described previously^61,69–71^ for both SSM and IFM. Citrate synthase (CS)^69^ and electron transport chain enzyme activities were measured in mitochondrial samples treated with cholate: CI, complex I – rotenone-sensitive; CIII, complex III - antimycin A-sensitive decylubiquinol‐cytochrome c reductase; NCR, rotenone-sensitive NADH-cytochrome c reductase; SCR, antimycin A-sensitive succinate-cytochrome c reductase; NFR, NADH-ferricyanide reductase; SDH, succinate dehydrogenase; aconitase; CII, thenoyltrifluoroacetone(TTFA)‐sensitive succinate‐Q reductase; CII + Q, TTFA-sensitive complex II with exogenous coenzyme Q_1_. The donors and acceptors span specific regions of the ETC^69–71^. The ETC activity components were determined using biochemical kinetics principles: CIV activity was determined as a first-order reaction relationship, whereas the activity of the other ETC components was determined with a zero-order reaction relationship.

### Immunoblotting

Isolated SSM and IFM mitochondria from 28 weeks old GK or Wistar rats were diluted 1:3 in loading buffer (4% SDS, 20% glycerol, 160 mM dithioerythrol, 125 mM Tris-Cl (pH 6.8), bromophenol blue 0.004%). Samples were then sonicated for 3 min and subsequently heated for 10 minutes at about 76–78 °C for denaturation. Sample’s proteins were then separated by electrophoresis on 4–20% Mini-PROTEAN® TGX™ precast polyacrylamide gels (Biorad, Hercules, CA, USA), and then transferred on PVDF membranes, which were blocked for 2 hours with 5% milk in Tris buffered saline with 0.1% Tween 20 (TBS-T). Then incubation with primary antibodies followed overnight at 4 °C. The following antibodies were used: anti-Aconitase-2 rabbit polyclonal antibody (Proteintech, dilution 1:1000); anti-COX IV rabbit polyclonal antibody (Invitrogen, dilution 1:1000). Secondary antibodies (goat anti-rabbit peroxidase conjugate 1:2000, Sigma-Aldrich) were incubated for 1 hour at room temperature. Detection of protein signals was achieved by using the ECL Prime chemiluminescence kit (GE Healthcare) and images acquisition using a Fujifilm Luminescent Image Analyzer LAS4000 System (Fujifilm, Tokyo, Japan). Immunoreactive bands were analyzed for densitometry with Image Studio Lite Software (LI-COR, Nebraska, USA). Proteins quantification was expressed as the relative intensity of protein signals normalized to the expression of the housekeeping gene COX IV.

### Statistical analysis

The results are reported as means ± standard deviation. Differences between control and diabetic rats at different ages were evaluated with two-way ANOVA with Bonferroni-Holm correction for multiple comparisons. Differences between control and diabetic rats at different time points of the insulin test were estimated with a two-tailed student t-test. A regression analysis was performed to determine whether a correlation exists between a mitochondrial marker enzyme (i.e. CS and SDH) and respiration rate. Differences were considered statistically significant at p < 0.05.

## Supplementary information

Supplementary information
