# Supplementary material for: Bioenergetic functions in subpopulations of heart mitochondria are preserved in a non-obese type 2 diabetes rat model (Goto-Kakizaki)
Source: Sci Rep. 2020 Mar 25;10:5444. doi: 10.1038/s41598-020-62370-8 (PMC7096416; doi:10.1038/s41598-020-62370-8)
Supplement: Supplementary file 1 — Supplementary information [file 41598_2020_62370_MOESM1_ESM.pdf]

Bioenergetic functions in subpopulations of heart mitochondria are preserved in a non-obese type 2 diabetes rat model (Goto-Kakizaki)

Lai N[1-3,5,7], Kummitha CM[3], Loy F[8], Isola R[8], Hoppel CL[4-6]

[1]*Department of Electrical and Computer Engineering;* [2]*Biomedical Engineering Institute; Old Dominion University, Norfolk, Virginia.* [3]*Department of Biomedical Engineering,* [4]*Department of Pharmacology;* [5]*Center for Mitochondrial Disease;* [6]*Department of Medicine, School of Medicine, Case Western Reserve University* [7]*Department of Mechanical, Chemical, and Materials Engineering and* [8]*Department of Biomedical Sciences, University of Cagliari*

Keyword: Heart mitochondria, Fatty Acid oxidation, Diabetes, Oxidative Phosphorylation

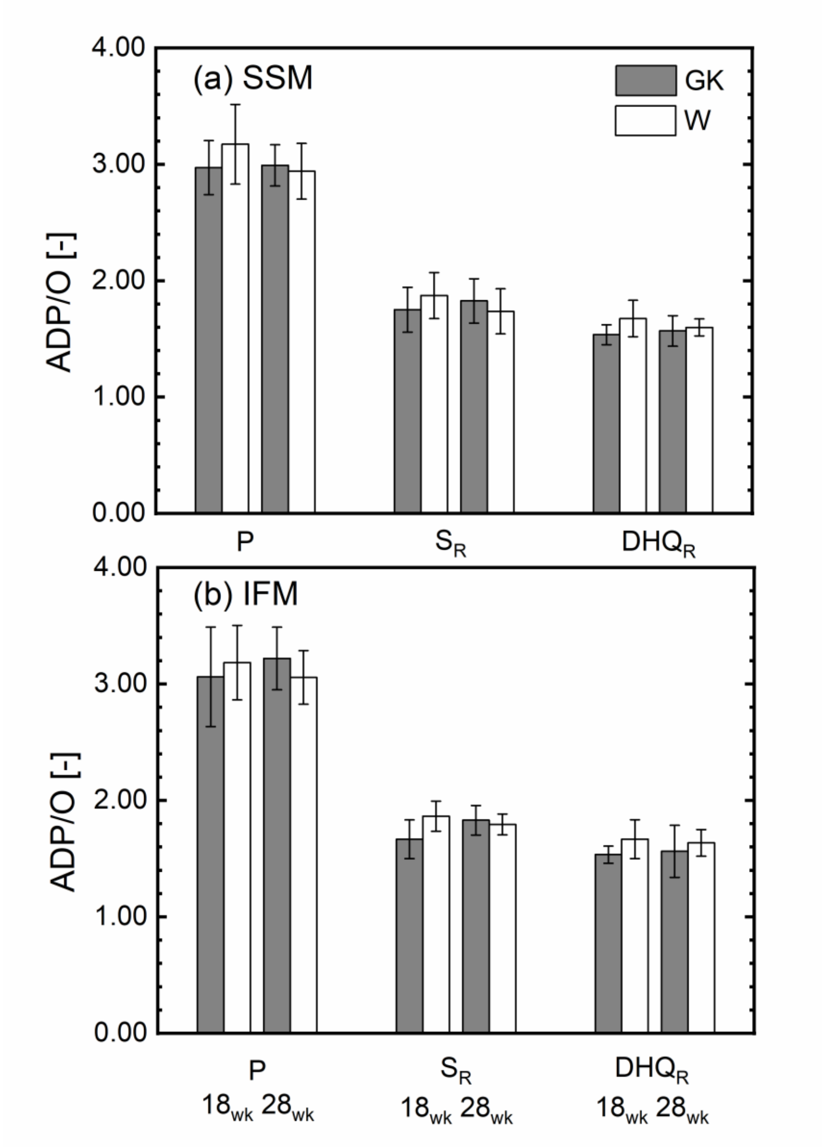

Fig. S1. ADP to atomic oxygen phosphorylation ratio ADP/O of heart muscle SSM (a) and IFM (b) at 18 and 28 weeks. Notation as in Fig 2. Complex I substrate (malate and pyruvate, P); Complex II (succinate and rotenone, S_R); Complex III (duroquinol and rotenone, DHQ_R). (n=6), Mean ± SD.

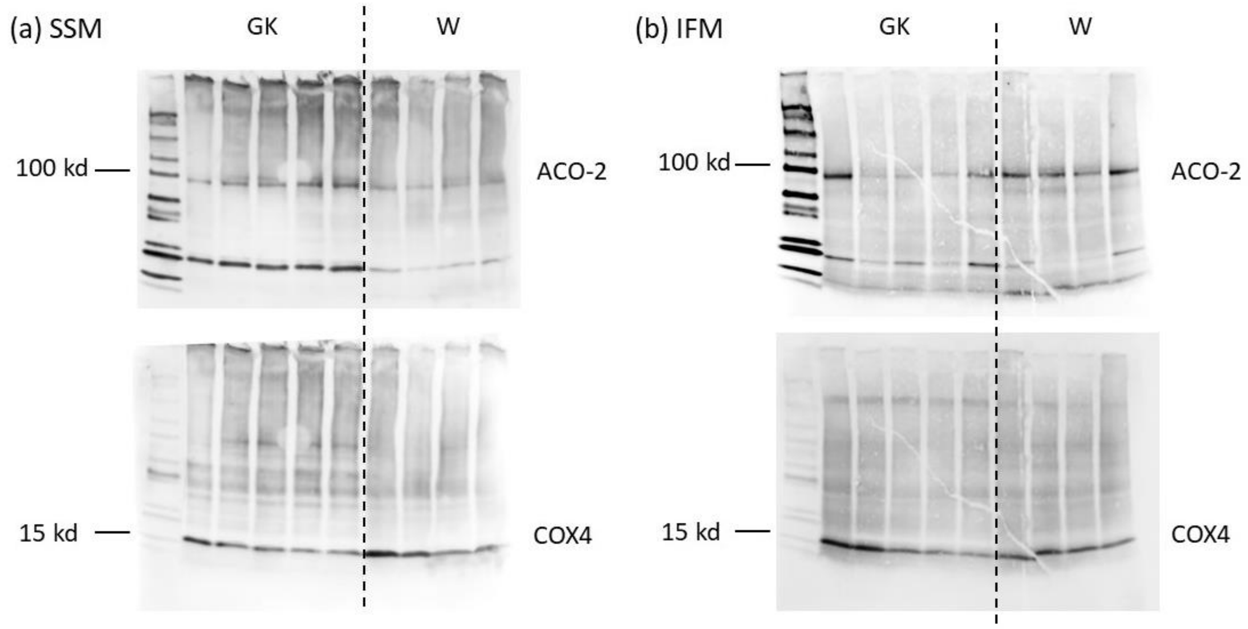

Fig. S2. Full-length immunoblotting of aconitase (ACO-2) protein in heart muscle SSM (a) and IFM (b) of Control (W) and diabetic (GK) groups at 28 weeks. Aconitase (ACO-2) and the housekeeping gene COX4 proteins are located at 85 and 17 kd, respectively. Cropped blots are reported in Figure 2 of the main text.
